# Computational Insights into Allosteric Conformational Modulation of P-Glycoprotein by Substrate and Inhibitor Binding

**DOI:** 10.3390/molecules25246006

**Published:** 2020-12-18

**Authors:** Juan Xing, Shuheng Huang, Yu Heng, Hu Mei, Xianchao Pan

**Affiliations:** 1 College of Basic Medical Science and College of Pharmacy, Southwest Medical University, Luzhou 646000, China; xingjuan217@163.com; 2 Key Laboratory of Biorheological Science and Technology (Ministry of Education), College of Bioengineering, Chongqing University, Chongqing 400045, China; shhuang@cqu.edu.cn (S.H.); 201819021102@cqu.edu.cn (Y.H.); meihu@cqu.edu.cn (H.M.)

**Keywords:** P-glycoprotein, ABC transporter, conformational changes, molecular dynamics

## Abstract

The ATP-binding cassette (ABC) transporter P-glycoprotein (P-gp) is a physiologically essential membrane protein that protects many tissues against xenobiotic molecules, but limits the access of chemotherapeutics into tumor cells, thus contributing to multidrug resistance. The atomic-level mechanism of how substrates and inhibitors differentially affect the ATP hydrolysis by P-gp remains to be elucidated. In this work, atomistic molecular dynamics simulations in an explicit membrane/water environment were performed to explore the effects of substrate and inhibitor binding on the conformational dynamics of P-gp. Distinct differences in conformational changes that mainly occurred in the nucleotide-binding domains (NBDs) were observed from the substrate- and inhibitor-bound simulations. The binding of rhodamine-123 can increase the probability of the formation of an intermediate conformation, in which the NBDs were closer and better aligned, suggesting that substrate binding may prime the transporter for ATP hydrolysis. By contrast, the inhibitor QZ-Leu stabilized NBDs in a much more separated and misaligned conformation, which may result in the deficiency of ATP hydrolysis. The significant differences in conformational modulation of P-gp by substrate and inhibitor binding provided a molecular explanation of how these small molecules exert opposite effects on the ATPase activity. A further structural analysis suggested that the allosteric communication between transmembrane domains (TMDs) and NBDs was primarily mediated by two intracellular coupling helices. Our computational simulations provide not only valuable insights into the transport mechanism of P-gp substrates, but also for the molecular design of P-gp inhibitors.

## 1. Introduction

The ATP-binding cassette (ABC) transporter P-glycoprotein (P-gp, also known as MDR1 or ABCB1) is a membrane-bound efflux pump, which can harness the energy of ATP hydrolysis to transport a wide variety of molecules out of diverse cells and many blood–organ barriers [1]. This membrane transporter thus plays important roles not only in protecting tissues from toxic xenobiotics and endogenous metabolites, but also in determining the pharmacokinetic parameters of commonly used drugs [2]. The US Food and Drug Administration (FDA) recommended that the interactions between P-gp and candidate drugs should be checked early in the drug discovery pipeline, with the aim to improve the pharmacological parameters and prevent the undesired off-target toxicity [3]. Most importantly, P-gp has a notorious function in powering the efflux of structurally and chemically unrelated anticancer agents, resulting in multidrug resistance which is a major clinical impediment to an effective cancer treatment [4]. Despite showing promise in cell- and animal-based models, the chemosensitization of drug-resistant cancer through coadministration of P-gp inhibitors with chemotherapeutics has not yet been clinically successful. As an important pharmacological target, it is indispensable to unravel the molecular basis of substrate transport by P-gp.

At the structural level, P-gp is a ~170-kDa molecule comprised of two pseudosymmetric halves, each containing a transmembrane domain (TMD) and a cytoplasmic nucleotide-binding domain (NBD). The TMDs determine the substrate specificity and form the transportation pathway by alternating between inward- and outward-facing orientations, while the dimerization and disengagement of NBDs, in turn, can drive the conformational switch of the TMDs, a process which is controlled by ATP binding and hydrolysis [5]. To facilitate the development of specific inhibitors for clinical practice, structural and dynamic insights into the P-gp transport and inhibition are indispensable. It has been proved that the ATPase activity and transport cycle of this efflux pump can be either simulated by drug substrates (e.g., paclitaxel and doxorubicin) or modulated by some inhibitors (e.g., tariquidar and cyclosporin A) [6]. As yet, the molecular mechanisms of how the substrate and inhibitor binding in TMDs is allosterically coupled to the ATP hydrolysis in NBDs and how these small-molecule compounds exert opposite effects on the transport cycle remain to be elucidated. Therefore, it is useful to compare the binding sites of substrates and inhibitors, and their respective effects on the conformational dynamics of P-gp.

The current molecular understanding of drug efflux by P-gp is largely dependent on the crystal structures, which can provide static snapshots that may play an important role in the conformational cycle for substrate transport. Several crystal structures in combination with biochemical data have revealed an intrinsically large, flexible, and hydrophobic binding pocket in TMDs for the binding of transport substrates and inhibitors [7,8,9,10,11]. A concern for these studies is that they were performed with the presence of detergents, a specific inhibitory antibody, and engineered disulfide cross-linking, which may have large effects on the conformational dynamics of P-gp under physiological conditions. Of note, the mechanism of P-gp is intrinsically dynamic. In particular, the molecular details of the conformational dynamics of P-gp modulated by small molecules still remain poorly understood at the atomic level. Additionally, ligand-based quantitative structure-activity relationship (QSAR) models have been well established in our previous researches for the prediction of P-gp substrates/non-substrates and inhibitors/non-inhibitors, respectively [12,13]. Combined with structure-based molecular docking, the binding sites and binding modes of substrates and inhibitors have been well characterized. However, the effects of these molecules on the dynamics of P-gp remain largely unknown.

Atomistic molecular dynamics (MD) simulations can provide structural and dynamic information under near-native conditions and hence, appear specifically well-suited for the mechanism research of membrane transporters [14]. In the previous work, we performed MD simulations with an enhanced sampling algorithm to investigate the entrance gate for the access of substrates or inhibitors from the lipid membrane to the binding pocket [15]. The aim of this work is to characterize at the atomic level the effects of the binding of a transport substrate and an inhibitor on the conformational transitions of P-gp by unbiased MD simulations in an explicit membrane/water environment. The results provided not only valuable insights into the transport mechanism of P-gp substrates, but also the molecular design of P-gp inhibitors.

## 2. Results and Discussion

### 2.1. Different Conformational Changes Mainly Occurred in the NBDs Upon Substrate and Inhibitor Binding to the TMD Cavity

**Figure 1:**
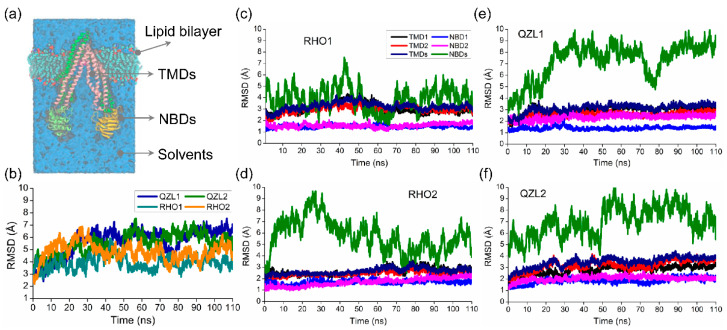
Time evolution of C_α_ root-mean-square deviations (RMSDs) across all simulations. (**a**) The starting structure with P-glycoprotein (P-gp) inserted in a solvated lipid bilayer. (**b**) The RMSDs of the overall structures. (**c**–**f**) The RMSDs of individual domains.

Two substrate-bound (RHO1 and RHO2) and two inhibitor-bound (QZL1 and QZL2) MD simulations were performed for the membrane-embedded and solvated P-gp systems, respectively. The time evolution of the root mean squared deviations (RMSDs) of the C_α_ atoms were calculated to evaluate the structural drift of P-gp relative to the starting structures for all simulations. C_α_ RMSDs of the overall structures showed a rapid increase in the first 30 ns, followed by a stable fluctuation over the course of all simulations (Figure 1b), which indicated that all simulations reached equilibrium in the last 80 ns, and at equilibrium states, both of the RHO- and QZL-bound P-gp underwent large conformational changes. It can be seen that the TMDs presented a similar RMSD of ~3 Å in all simulations. By contrast, the RMSDs of the NBDs were much larger than those of TMDs, ranging from ~3 to ~9 Å across all the simulations (Figure 1c–f). This suggested that the conformational changes substantially occurred in the NBDs upon substrate and inhibitor binding. Prominently, the NBDs seemed to vacillate between several conformational states with substrate binding, which was discussed in the following sections. However, the RMSDs of the NBD monomers (NBD1 and NBD2) were almost less than 2 Å, indicating a lack of significant conformational changes. Therefore, it can be inferred that the major conformational changes of P-gp under substrate- and inhibitor-bound conditions were mainly triggered by the rigid-body movement between the two NBD monomers.

**Figure 2:**
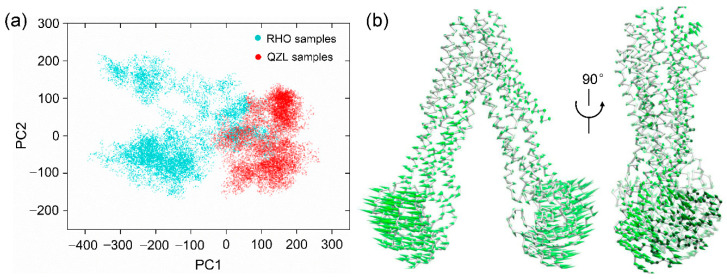
Principal component analysis (PCA) of equilibrium trajectories. (**a**) The distribution of conformational samples in the first two principal components. (**b**) Directions of motion along PC1 represented by porcupines. The average structure of P-gp was shown as ribbon. For clarity, the reverse direction was not shown.

Figure 2a showed the projection of conformational samples onto the subspace spanned by the first two principal components, PC1 and PC2. The points therein represented 16,000 snapshots sampled from MD trajectories. Notably, despite a little overlap existing, the samples can be mainly divided into two groups by PC1, which happened to be characterized by the RHO- and QZL-bound states, respectively. The structural variance along PC1 was dominated by the relative motion between NBD1 and NBD2, which can describe the predominant movement of the transporter (Figure 2b). These results were suggestive of a significant difference in the conformational distribution and conformational dynamics of NBDs when the substrate and inhibitor bound to P-gp.

### 2.2. Conformational Homogeneity in the Substrate- and Inhibitor-Bound States

**Figure 3:**
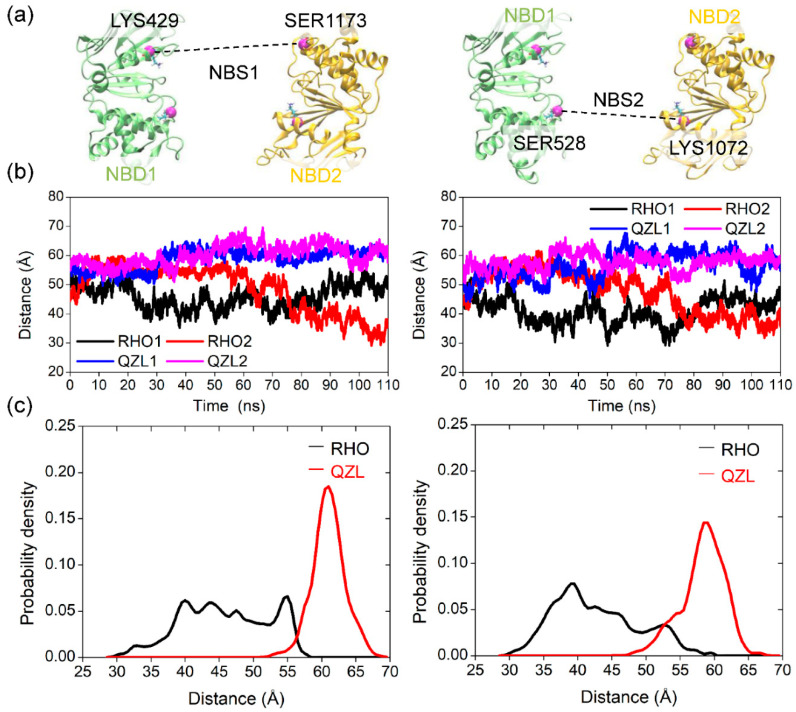
Distances between the two nucleotide-binding sites (NBSs) in the substrate- and inhibitor-bound states. (**a**) Cartoon representation of nucleotide-binding domains (NBDs) showing the conserved residue pairs at the NBSs across the dimer interface. The Cα atoms of the conserved residues were shown as purple spheres. (**b**) Time series of the distances between the conserved residues. (**c**) Distance distributions over all trajectories from the last 80 ns simulations under substrate- and inhibitor-bound conditions. The distributions were calculated every 0.5 Å.

To quantitatively depict conformational changes and conformational states under the substrate- and inhibitor-bound conditions, the C_α_–C_α_ distances in the nucleotide-binding sites (NBSs) between the conserved residues of the Walker A and signature motifs from opposite NBDs, which are important for ATP binding and hydrolysis were measured (Figure 3a). Significant changes of the distances in both NBSs were observed in the RHO- and QZL-bound simulations (Figure 3b), suggesting a large relative movement between NBD1 and NBD2. The shape of the distance distributions uncovered a fundamental conformational difference between substrate- and inhibitor-bound states (Figure 3c). The distance distributions in both sites were heterogeneous under the substrate-bound state, hinting at a conformational equilibrium comprising multiple conformations. The broad distance distributions also suggested that the substrate-bound P-gp was highly dynamic in the absence of nucleotides. In comparison with the initial structure, the pair distances in both sites showed a moderate decrease under the substrate-bound condition (Figure 3b,c), suggesting a closer tendency of the two NBDs upon substrate binding. Previous data from double electron-electron resonance spectroscopy (DEER), electron microscopy (EM), and MD simulation studies suggested a dynamic equilibrium of various conformers of the NBDs in the apo state, prevailing in a more open conformation than the crystal structure [16,17]. Therefore, despite multiple conformations accessed at the equilibrium state, the presence of a substrate may shift the conformational equilibrium to an intermediate that can increase the chance of formation of interfacial interactions between the two NBD monomers, in order to accelerate the dimerization process.

By contrast, binding of the inhibitor QZL induced a large relative movement that facilitated a wide-open NBD dimer, as indicated by the dramatic increase in both of the pair distances to around 60 Å (Figure 3b). Moreover, the narrower distance distributions demonstrated a restriction of conformational flexibility in the inhibitor-bound state (Figure 3c), which suggested that binding of an inhibitor may stabilize a single and homogeneous conformation distinct from that observed under substrate-bound conditions. Anyway, these observations were in line with the experimental data, which demonstrated that the two NBDs were disengaged in the presence of a transport substrate or an inhibitor [18].

### 2.3. Implications of Substrate- and Inhibitor-Coupled Conformational Transitions

**Figure 4:**
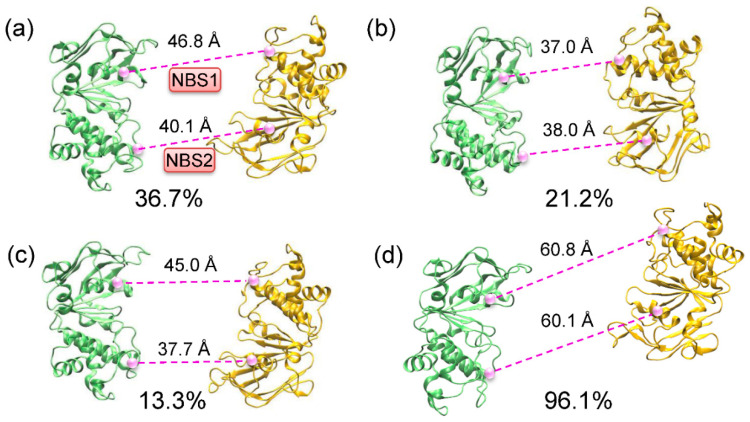
Clustering analysis of molecular dynamics (MD) samples of P-gp. (**a**–**c**) The representative conformations of NBDs from the first three conformational subsets in the RHO-bound state. (**d**) The represented conformation of NBDs in the QZL-bound state. The C_α_–C_α_ distances between the conserved residues of the Walker A motif and the signature motif were shown as dashed lines in the NBSs.

To further investigate the conformational transitions driven by substrate and inhibitor binding, the dominant populations and representative conformations were determined by the clustering analysis of the 160 ns (80 × 2) equilibrium trajectories of each state (Figure 4). In the substrate-bound state, the largest population (36.7%) resided in an apo-like conformation, in which the two NBDs were twisted relative to each other, as indicated by the uneven separations between the two NBSs (Figure 4a). A comparable portion of the conformational samples (34.5%) showed a relatively tightened conformation of NBDs (Figure 4b,c), suggesting that substrate binding was inclined to bring the NBDs nearer to each other. In ABC transporters, ATP hydrolysis occurred when the NBDs dimerized to form two complete catalytic sites. Here, the secondary population (21.2%) showed that both sites were about 37 Å apart, with the Walker A and signature motifs from opposite NBDs better in line with each other (Figure 4b). Consequently, the binding of rhodamine-123 in the TMDs may drive the NBDs to a pre-dimer configuration that can increase the rate of the dimerization of the NBDs and therefore, the formation of catalytically competent NBSs for ATP hydrolysis. This provides a molecular explanation of the elevated ATPase activity in the presence of a transport substrate. Moreover, the conformational changes in substrate-bound P-gp simulations were much smaller, which was consistent with the modest stimulation (2- to 16-fold) of ATP hydrolysis in P-gp [19]. Recently, Doshi and van Veen demonstrated that substrate binding to MsbA, a bacterial homologue of P-gp, initiated the dimerization of the NBDs and stabilized a pre-translocation intermediate for ATP binding [20]. Johnson and Chen determined the structures of apo and LTC4-bound multidrug resistance protein MRP1 [21]. A structural comparison showed that the NBDs were closer together in the substrate-bound state. Therefore, it can be inferred that the multidrug ABC transporters may share a common mechanism, in which the substrates stimulate ATP hydrolysis. However, the assembly of the hydrolysis-competent NBD dimer (the distances between the conserved motifs were ~10 Å) requires the synergetic binding of nucleotides, as observed by the outward-facing structures of the P-gp [22], MRP1 [23], and MsbA [24] in the ATP-bound state.

In contrast to the transport substrate, where multiple conformations likely coexisted, the inhibitor QZL stabilized a single conformation, in which the NBDs were much more separated (Figure 4d). The relative sliding movement between the two NBDs resulted in the misalignment of the Walker A and signature motifs from opposite NBDs. Thereby, we inferred that the binding of a small molecular inhibitor may impede the dimerization of NBDs, thus resulting in the deficiency of ATP hydrolysis. Our data are compatible with the proposed mechanism that drug binding in the TMDs can induce long-range conformational changes in the NBDs to stimulate or inhibit ATP hydrolysis, through decreasing or increasing the distance between the Walker A and signature motif from opposite NBDs [25].

**Figure 5:**
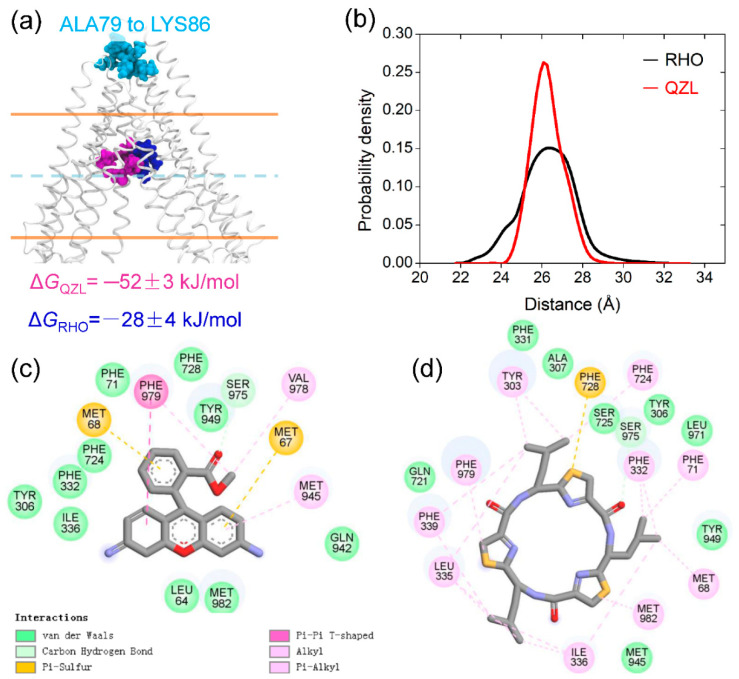
Binding sites in the upper transmembrane domain (TMD) cavity. (**a**) Cartoon representation of TMDs showing the overlap of RHO (blue) and QZL (purple). The reference residues (ALA79 to LYS86) at the extracellular side were shown as spheres (cyan). The lipid bilayer was indicated by orange lines. (**b**) Distance distribution between the center of masses of the compound and reference residues. (**c**,**d**) Interactions of RHO and QZL with the residues in the TMD cavity obtained by the decomposition of their binding free energies, respectively.

Furthermore, the binding sites of RHO with a relatively low affinity (Δ*G*_RHO_ = −28 ± 4 kJ/mol) and the high-affinity QZL (Δ*G*_QZL_ = −52 ± 3 kJ/mol) were highly overlapped in the upper TMD cavity (Figure 5a,b). A key subset of residues contributing to the binding and transport of rhodamine dyes has been identified by site-directed mutagenesis [26,27,28], including Leu64, Phe332, Ile336, Phe724, Tyr949, Val978, and Met982 (Figure 5c). Previous MD simulations also suggested that binding of a substrate in the upper site may promote the NBD closure [29]. The interactions of QZL in the upper site with aromatic residues that have been implicated in the opening of the TMD cavity to the outside (Figure 5d), thus possibly stabilized the inward-facing state [30]. Accordingly, it can be further speculated that the binding of an inhibitor may block the access for a substrate, prevent the conformational changes required for substrate release and ATP hydrolysis, and eventually inhibit the drug transport cycle. Intriguingly, Loo and Clarke proposed a distinct inhibition mechanism for a unique P-gp inhibitor, tariquidar, which inhibited the drug efflux by blocking the conformational transition of P-gp from a closed to open state during the catalytic cycle [31,32]. This suggested a mechanistic divergence in P-gp inhibition by the binding of inhibitors with different chemical structures.

### 2.4. Allosteric Communication between TMDs and NBDs upon Substrate Binding

**Figure 6:**
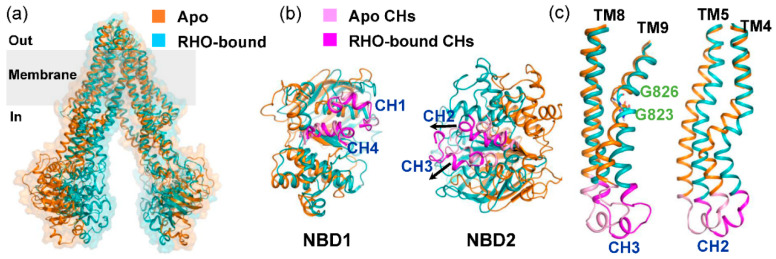
Conformational changes upon rhodamine-123 binding. (**a**) Global superposition of the apo (PDB code: 4Q9H) and RHO-bound structures. The RHO-bound snapshot was the representative frame taken from the second populated cluster, as shown in Figure 4b. (**b**) Extracellular view of the NBDs after the structural superposition of TMDs. Arrows indicate movements of CHs. (**c**) Close-up view of the TM helix pairs TM4-TM5 and TM8-TM9. Two glycine residues located on TM9 were displayed as sticks.

An open question at present is how the substrate-induced conformational changes within TMDs propagate to the cytoplasmic NBDs. The structural comparison of the apo and RHO-bound P-gp revealed that both global and local conformational changes were induced by substrate binding (Figure 6). It can be observed that substrate binding facilitated the contraction of TMDs near the cytosol, although the RHO-bound simulations showed minor changes in the TMD cavity (Figure 6a). In P-gp and other ABC exporters, the intracellular coupling helices (CHs), each of which was formed by two TMs mediate physical connections to the NBDs. In the case of substrate binding, there were a number of small but notable displacements localized primarily in the CH2 and CH3, leading to the reorientation of NBDs to a pre-dimer configuration (Figure 6b). Cross-linking showed that the conformational flexibility of CH2/CH3 and the hydrophobic network at the CH2/CH3/NBD2 transmission interface were critical for the transport activity of P-gp [33]. A further structural analysis revealed that the conformational transition from the apo state to the RHO-bound state included some non-negligible changes in the TMDs, most significantly in the intracellular conformations of TM4 and TM9 (Figure 6c). The two glycine residues increased the flexibility of TM9, thus allowing for the helix bending motion. The flexibility and ligand-induced movement of TM4 have been observed from the crystal structures, which is also critical for drug binding and transport [9,30]. Together, it is suggested that the substrate-induced conformational changes in TM4 and TM9 may provide an allosteric communication with the NBDs through CH2 and CH3.

Recently, a cryo-electron microscopy structure of substrate-bound P-gp reconstituted in lipidic nanodiscs has been determined by the use of a conformation-selective inhibitory antibody [34]. The structure showed that TM4 and TM10 adopted a kinked conformation, with Taxol bound in the enclosed TMD cavity. The two helix breaks pinched the TMD cavity by increasing substrate interactions and closed the intracellular gate, thereby decreasing the NBD-NBD distances. However, these conformational changes were not observed in our work. One possible explanation is that the kinked and occluded conformation, as presented in the Taxol-bound P-gp, is a rather deep free energy minimum and the simulations are thus kinetically trapped on the nanosecond time scale. An alternative explanation could be that the structurally and physicochemically diverse substrates interacting with P-gp are likely to cause different conformational changes in TMDs by the induced-fit mechanism [11]. A detailed comparison of these differences would need to build various substrate-bound P-gp models and simulate them on a large time scale, which is beyond the scope of this work.

## 3. Materials and Methods

### 3.1. Structural Preparation

#### 3.1.1. Construction of Inhibitor- and Substrate-Bound P-gp Models

The corrected X-ray crystallographic structures of murine P-gp in the apo (ligand free) state, 4Q9H, and in the inhibitor-bound state, 4Q9K, were retrieved from the RCSB Protein Data Bank [35], which were characterized by an inward-facing conformation with relatively high resolutions (3.4 and 3.8 Å, respectively). The cyclopeptide inhibitor QZ-Leu (QZL) was extracted from 4Q9K and then docked into 4Q9H. Both of the co-crystallized and the docked structures of P-gp with QZL occupied at the binding cavity were used as the initial model to reduce the risk of bias and were stochasticity introduced by using a single conformation. Then, the commonly used fluorescent probe substrate rhodamine-123 (RHO) was docked into the binding cavity of 4Q9H and 4Q9K, respectively. The resultant structures were used as the initial model for substrate-bound simulations. Hereafter, the RHO– and QZL–bound simulations for 4Q9K were referred to as RHO1 and QZL1, while the others for 4Q9H were referred to as RHO2 and QZL2, respectively.

#### 3.1.2. Molecular Docking

Molecular docking was performed using the Surflex-Dock [36] protocol built-in Sybyl 8.1 package (Tripos Inc., St. Louis, MO, USA), as described earlier [12]. Briefly, the conformation search was guided by an ensemble of small probes (CH_4_, NH, and CO) referred to as a “protomol”, which can make favorable interactions with a predefined binding site. The residues of TMDs facing the internal cavity were selected to generate the “protomol” using a thresh of 0.5 and a bloat of 5. The flexibility of side chains within a 4 Å distance from a ligand was allowed to adapt the conformation of the docked ligand. The best scored docking poses were used to construct the starting structures for the following MD simulations.

#### 3.1.3. Preparation of Simulation Systems

The orientation and insertion depth of P-gp in the lipid bilayer was determined with OPM (orientation of proteins in the membrane database). Each P-gp model was inserted in a lipid bilayer composed of 366 1-palmitoyl-2-oleoyl-*sn*-glycero-3-phosphocholine (POPC) lipid molecules using the CHARMM-GUI membrane builder [37], solvated by TIP3P [38] water molecules on each side of the membrane, and then neutralized by an adequate number of Cl^−^ ions. A concentration of 0.15 M NaCl was added to mimic the physiological ionic condition. The resulting system contains approximately 244,000 atoms with a box dimension of 120 × 120 × 180 Å (Figure 1a). All basic residues in the protein were positively charged, and acid residues were negatively charged. As suggested by O’Mara and Mark [39], all histidines (His) were protonated at the *ε* nitrogen atoms, with the exception of His149, His583, and His1228, in which both *δ* and *ε* nitrogen atoms of the imidazole ring were protonated. The N- and C-terminal residues of the protein were modeled as neutral.

### 3.2. MD Simulations

#### 3.2.1. Simulation Parameters

MD simulations were performed using the Amber 18 suit of programs [40]. The protein, POPC, and ligands were parameterized using the AMBER ff14SB force field [41], Lipid14 [42], and general amber force field (GAFF) [43], respectively. The atomic partial charges of the ligands were calculated using the AM1-BCC model [44]. The RHO was processed with one positive charge. An integration time step of 2 fs was used for all simulations. The semi-isotropic Berendsen coupling barostat with compressibility of 4.5 × 10^−5^ bar^−1^ was applied to equilibrate the pressure by separately coupling the lateral (*xy*) and normal (*z*) box directions, whereas the Langevin thermostat was used to equilibrate the temperature with a collision frequency of 1.0 ps^−1^. Long-range electrostatic interactions were estimated using the partial mesh Ewald (PME) method [45] with an interpolation order of 6, and bonds involving hydrogen atoms were constrained using the SHAKE algorithm [46]. A nonbonded cutoff of 10 Å was used for the van der Waals and electrostatic interactions.

#### 3.2.2. Simulation Protocol

Prior to MD simulations, each system was energy-minimized for 5000 steps (2500 steps of the steepest descent and 2500 steps of conjugate gradient minimization) with 50 Kcal·mol^−1^·Å^−2^ harmonic-position restraints applied to the protein, ligand, and lipids, followed by another 5000 steps of the same algorithm with the protein and ligand restrained by a harmonic force of 10 Kcal·mol^−1^·Å^−2^, ending with 10,000 steps with no restraints. Then, the system was heated linearly to 100 K by 20 ps constant volume dynamics and then slowly to 310 K by 120 ps constant pressure dynamics using a harmonic restraint of 10 Kcal·mol^−1^·Å^−2^ on all atoms of the protein, ligand, and lipids. Next, the lipid species was pre-equilibrated by 2 ns dynamics with the protein, ligand, and phosphorus atoms restrained by a force constant of 1 Kcal·mol^−1^·Å^−2^, followed by another 2 ns dynamics by removing the harmonical restraints on the phosphorus atoms.

Subsequently, ten rounds of restrained MD simulations were performed at a constant temperature and pressure (*T* = 310 K; *P* = 1 bar), allowing for the relaxation of the lipids and water molecules around the protein and optimization of the ligand interactions with the protein sidechains. In this step, the restraints on the protein Cα atoms were gradually decreased from 5 to 0.1 Kcal·mol^−1^·Å^−2^ over 20 ns. Finally, an unbiased production simulation of 110 ns in length was performed in an NPT ensemble (*T* = 310 K; *P* = 1 bar) for each of the refined system.

### 3.3. Trajectory Analysis

#### 3.3.1. Principal Component Analysis

The principal component analysis (PCA) was performed to unveil the conformational differences and dominant motion modes of P-gp. First, a sample set including 16,000 snapshots was retrieved from the last 80 ns trajectories of each system, and used to calculate the average structure. After removing the overall translations and rotations by the alignment of all snapshots with the average structure, a 3*N* × 3*N* (*N* is the number of Cα atoms) covariance matrix ***C*** was constructed based on the Cα coordinates, and ***C*** was then diagonalized to determine the eigenvalues and eigenvectors (principal components, PCs), which can describe the principal modes of structural variations. The principal modes were rank-ordered: PC1, corresponding to the direction of the largest variance, was succeeded by PC2, etc. Generally, the first two principal axes, PC1 and PC2, can account for most of the total variance in the structure. Therefore, the projection of dataset structures onto the subspace spanned by PC1 and PC2 can be used to discriminate or cluster the conformations based on their most distinctive structural similarities and/or dissimilarities. PCA was carried out using the Cpptraj module of Amber 18.

#### 3.3.2. Cluster Analysis

To determine the dominant populations and representative conformations, the equilibrium trajectories of each state were clustered using the Cpptraj module of Amber 18. Two conformations of NBDs were considered to fall into the same cluster if the Cα RMSD between the conformations was less than 1.5 Å.

### 3.4. Free Energy Calculations

The free energies of RHO and QZL binding to P-gp were estimated by the MM/GBSA (molecular mechanics/generalized born surface area) method based on 1600 snapshots obtained from the equilibrium states. The free energy calculations were performed by the Python script MMPBSA.py built-in Amber 18. POPC lipids, explicit water molecules, and ions were stripped from the trajectories. The lipid membrane was treated implicitly in MM/GBSA calculations. The dielectric constants of the protein and implicit solvent were set to 2 and 80, respectively. The salt concentration was set to 0.15 M. It should be emphasized that the calculated free energies were not expected to be exact, but rather to show a relative likelihood of the ligand binding.

## 4. Conclusions

In summary, we investigated the effects of small-molecule compounds on the conformational transitions of the prevalent multidrug transporter P-gp, using all-atom MD simulations in an explicit membrane and water environment. Primary conformational changes in the relative orientation between the two NBDs were observed. The substrate-bound simulations accessed a conformation, in which the NBDs were closer and better aligned, suggesting that substrate binding may prime the transporter for ATP hydrolysis. By contrast, the inhibitor stabilized the NBDs in a much more separated and misaligned conformation, which may impair ATP hydrolysis. The results suggested that the dimerization of NBDs and the formation of the catalytic sites for ATP hydrolysis could be differentially modulated by the binding of substrates and inhibitors, which can further affect the transport function of P-gp. The conformational changes of TM helices induced by substrate binding can propagate to NBDs through the CHs located at the TMD-NBD transmission interface. In addition to providing atomic-level insights into the molecular mechanism of drug-coupled ATP hydrolysis by P-gp, the short MD simulations could be used to identify potential modulators for multidrug transporters in general, thereby accelerating the development of specific inhibitors for clinical use. In the future, delineation of the complete conformational cycle of P-gp at the atomic level is the next central question.

## Abbreviations

P-gp	P-glycoprotein
TMDs	transmembrane domains
NBDs	nucleotide-binding domains
ABC	ATP-binding cassette
NBSs	nucleotide-binding sites
MD	molecular dynamics
RMSDs	root-mean-square deviations
PCA	principal component analysis
MM/GBSA	molecular mechanics/generalized born surface area
